# Supplementary material for: Mesenchymal Stem Cell-Derived Extracellular Vesicles Protect Human Corneal Endothelial Cells from Endoplasmic Reticulum Stress-Mediated Apoptosis
Source: Int J Mol Sci. 2021 May 6;22(9):4930. doi: 10.3390/ijms22094930 (PMC8125791; doi:10.3390/ijms22094930)
Supplement: Supplementary file 1 [file ijms-22-04930-s001.zip › ijms-1182400-supplementary.pdf]

Supplementary Material

Mesenchymal Stem Cell-Derived Extracellular Vesicles Protect Human Corneal Endothelial Cells from Endoplasmic Reticulum Stress-Mediated Apoptosis

Lola Buono, Simona Scalabrin, Marco De Iuliis, Adele Tanzi, Cristina Grange, Marta Tapparo, Raffaele Nuzzi and Benedetta Bussolati

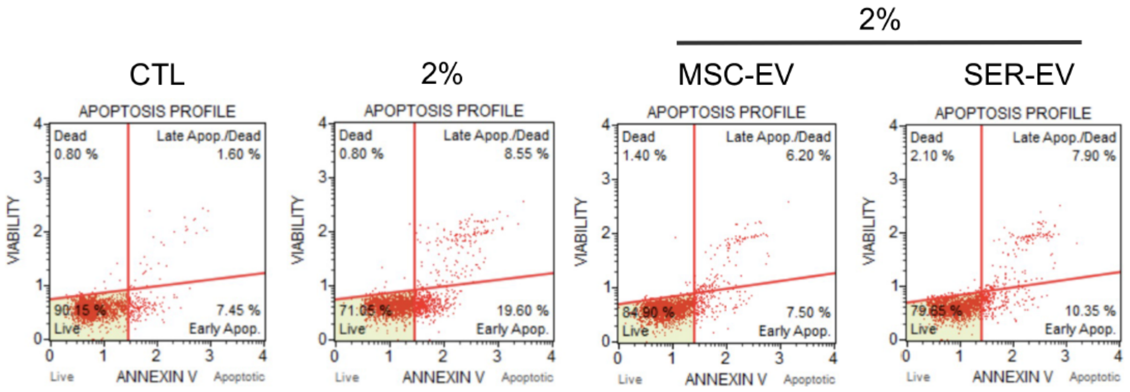

Figure S1. Representative dot plots showing viability (Dead Cell Marker 7-AAD) vs. apoptosis (Annexin V) of stained HCECs. Cells were untreated (CTL) or under serum deprivation (2%) with or without MSC-EVs or SER-EVs (20×10^3/target cell). The percentages of each population are reported: lower-left quadrant: viable cells; lower- right quadrant: cells in the early stages of apoptosis; upper-right quadrant: cells in the late stages of apoptosis or dead; upper-left quadrant: cells that have died not through the apoptotic pathway.

Table S1. Clinical and biological information of patients undergoing penetrating keratoplasty. Clinical and biological aspects of patients from which we received corneal buttons. FECD: Fuchs' Endothelial Corneal Dystrophy; PKP: Penetrating Keratoplasty.

Sex	Age	Diagnosis	Surgical Procedure	Ophtalmological Therapy
F	75	Ocular hypertonia of traumatic etiology	Enucleation	Topical therapy: timolol, diclofenac
F	35	Keratoconus	PKP	Topical therapy: hydrocortisone
F	66	FECD	PKP	Topical therapy: loteprednol
F	47	Corneal leukoma	PKP	
M	75	Corneal leukoma	PKP	Systemic therapy: acetazolamide<br>Topical therapy: brinzolamide, timolol
M	78	Corneal leukoma	PKP	
F	83	FECD	PKP	Topical therapy: indomethacin, bromfenac, edenorm
F	47	FECD	PKP	
M	81	FECD	PKP	Topical therapy: netilmicin, dexamethasone, ofloxacin
M	40	Keratoconus	PKP	
F	40	Pellucid marginal degeneration	PKP	
F	77	FECD	PKP	Topical therapy: brinzolamide, timolol, brimonidine
M	80	FECD	PKP	Topical therapy: chloramphenicol dexamethasone, bluyal a
F	70	Corneal leukoma	PKP	
F	72	Corneal leukoma	PKP	Topical therapy: trehalose, clobetasone
F	31	Keratoconus	PKP	
M	71	FECD	PKP	

M	67	FECD	PKP
F	24	Corneal leukoma	PKP
M	75	Transplanted cornea late failure	PKP
M	78	Transplanted cornea late failure	PKP
F	21	Corneal leukoma (chemical burn)	PKP
F	66	Corneal leukoma	PKP

Table S2. Primer sequence list for mRNAs and miRNAs tested in the study. 1–5, primers for mRNA detection, 6–11 primers for miRNA detection.

	Symbol	Forward	Reverse
1.	GAPDH	TGGAAGGACTCATGACCACAGT	CATCACGCCACAGTTTCCC
2.	ATF4	TTCTCCAGCGACAAGGCTAAGG	CTCCAACATCCAATCTGTCCCG
3.	GRP78	CTGGCAAGATGAAGCTC	GGAGTGAAGGCGACATAGGA
4.	XBP1	GGTCTGCTGAGTCCGCAGCAGG	AGTTCATTAATGGCTTCCAGCT
5.	CHOP	TCTGGTCTTGTGGGGTATGC	AAAGCTGAGTTGGCCAGGAC
6.	RNU6B	CGCAAGGATGACACGCAA	
7.	hsa-miR-222-3p	AGCTACATCTGGCTACTGGGT	
8.	hsa-miR-125b-5p	TCCCTGAGACCCTAACTTGTG	
9.	hsa-miR-21-5p	TAGCTTATCAGACTGATGTTG	
10.	hsa-miR-214-3p	ACAGCAGGCACAGACAGG	
11.	hsa-miR-199a-3p	CCCAGTGTTCAGACTACCTGTTC	
